# Cosexuality Reduces Pollen Production and Fitness in *Cannabis sativa* L.

**DOI:** 10.3390/plants12213731

**Published:** 2023-10-31

**Authors:** Sydney B. Wizenberg, Jillian Muir-Guarnaccia, Lesley G. Campbell

**Affiliations:** 1 Department of Chemistry and Biology, Toronto Metropolitan University, 350 Victoria St, Toronto, ON M5B 2K3, Canada; sydneybw@yorku.ca (S.B.W.);; 2 Department of Biology, York University, 4700 Keele Street, Toronto, ON M3J 1P3, Canada

**Keywords:** *Cannabis sativa*, dioecy, sexual lability, cosexual, phenology, pollen

## Abstract

*Cannabis sativa* L. is cultivated globally for its cannabinoid-dense inflorescences. Commercial preference for sinsemilla has led to the development of methods for producing feminized seeds through cross-pollination of cosexual (masculinized) female plants. Although the induction of cosexuality in *Cannabis* plants is common, to date, no work has empirically tested how masculinization of female *Cannabis* plants impacts male flowering, pollen production, pollen fitness, and related life-history trade-offs. Here, we cultivated a population of *Cannabis* plants (CFX-2) and explored how the route to cosexuality (drought vs. chemical induction) impacted flowering phenology, pollen production, and pollen fitness, relative to unsexual male plants. Unisexual males flowered earlier and longer than cosexual plants and produced 223% more total pollen (F_2,28_ = 74.41, *p* < 0.001), but per-flower pollen production did not differ across reproductive phenotypes (F_2,21_ = 0.887, *p* = 0.427). Pollen viability was 200% higher in unisexual males and drought-induced cosexuals (F_2,36_ = 189.70, *p* < 0.001). Pollen non-abortion rates only differed in a marginally significant way across reproductive phenotypes (F_2,36_ = 3.00, *p* = 0.06). Here, we demonstrate that masculinization of female plants impacts whole-plant pollen production and pollen fitness in *Cannabis sativa*.

## 1. Introduction

*Cannabis sativa* L. (Cannabaceae) is sexually labile, possessing the ability to flexibly transition from female or male reproductive function to a cosexual phenotype that produces both ovules and pollen grains [1,2,3,4,5]. Cosexual plants are naturally occurring in *C. sativa*, often seen in response to stressful external environments [3,4,5], but they can also be synthetically induced through application of chemical treatment [6,7,8,9,10]. Manipulation of *C. sativa*’s sexual expression (a process known as sexual lability) has given rise to the production of feminized seeds. When a genetically female plant with an XX karyotype produces pollen, all gametes produced possess X chromosomes, and thus, the resulting offspring, if mating with another genetically female plant, all possess XX karyotypes [6]. Mass production of feminized seeds has been revolutionary to the cannabis industry due to its commercial emphasis on sinsemilla, unpollinated female inflorescences. Yet, to date, no work has explored how cosexuality impacts pollen production and fitness in this widely cultivated crop.

Cosexuality provides an opportunity for an individual to invest in both male and female reproduction [4,11]. In addition to the reproductive assurance provided by the ability to self-pollinate, cosexual plants benefit from the opportunity to be successful at both male and female reproduction separately [11,12,13,14,15,16], potentially increasing their contribution to the progeny gene pool. Cosexual plants are capable of mating with any individual in a population, removing previous reproductive barriers imposed by production of a single type of gamete, and increasing their ability to provide progeny with a diverse gene pool via mating with multiple partners. Though cosexuality is broadly beneficial in its ability to increase reproductive success, plants that invest in both male and female reproduction are often limited in their capacity to do either [17,18,19,20]. In instances of fixed resource access, any investment into the production of one type of gamete must be performed at the expense of the other, limiting their output and facilitating the development of fitness trade-off’s [21,22,23]. A reduced capacity to produce male gametes may be detrimental in populations that experience pollen limitation, providing optimal conditions for unisexual males to out-compete their cosexual competitors through mass production and dispersal of pollen grains. The competitive dynamics between unisexual and cosexual plants and their impact on the evolutionary trajectory of a population is interesting more broadly, but in particular, in a sexually labile crop like *C. sativa* that is cultivated for its floral biomass, understanding any factors that could impact reproductive characteristics may prove to be economically valuable.

*C. sativa* presents a rare and ideal model system for exploring these dynamics because it is both dioecious and labile in its sex expression. Dioecy is rare, but phylogenetically widespread [12], and in its most advanced form, dioecious plants may evolve sex chromosomes and rely on karyotype-based sex determination [1]. Karyotype-based sex determination is a form of genetic sex determination, wherein the presence or absence of a particular sex chromosome is often the defining feature in determining the development of primary sex characteristics. Karyotype-based determination of sex expression in plants generally takes one of two forms: Z/W chromosomal determination (female heterogamety), and X/Y chromosomal determination (male heterogamety) [1]. Within these sub-classifications, the evolutionary trajectory of these transitions can differ substantially [1], supporting the idea of multiple independent evolutionary events. In this sense, *C. sativa* being one of a few numbers of species on earth that is capable of flexibly producing both unisexual (dioecious) and cosexual (monecious) phenotypes [4], in addition to its reliance on X/Y karyotype-based sex determination [6], makes it an ideal study system for exploring these dynamics. This is compounded by its economic value as a crop cultivated globally for its reproductive biomass.

Prohibition on the growth and use of *C. sativa* throughout human history has inhibited our understanding of this economically valuable species [24,25,26,27,28]. Modern research has often focused on female plants [29,30,31,32,33], leaving a gap in our understanding of male plants, cosexual plants, male–male competition, and factors that impact male reproductive dynamics. Male–male competition is likely not limited to the adult life stage of male plants; the pollen grains they produce may interact with each other, representing a secondary stage of the reproductive competition and an additional opportunity for sexual selection to act [34,35,36,37,38]. The intergenerational nature of the plant life cycle could lead to differences in the direction and power of selection between paternal male plants and their haploid pollen grains [39,40,41,42], and in the case of the latter, the maintenance and expression of pollen-specific genes has implied that pollen grains are functionally independent organisms that act as vectors for male gametes [43]. Genotypic differences between pollen grains set the stage for differences in their competitive abilities and facilitate selection for pollen grains that possess increased fitness relative to pollen from other sires [43,44]. Therefore, any exploration of differences in reproductive performance and its potential contribution to sexual selection in plants must consider differences at the pollen grain stage of the plant life cycle.

**Figure 1:**
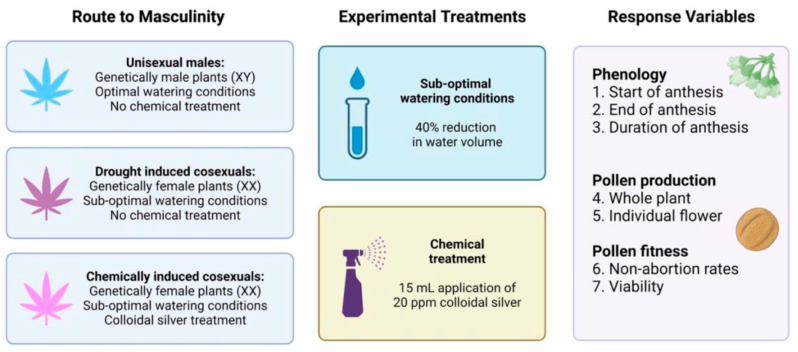
Conceptual image showing the experimental design. Each experimental group experienced one of three routes to masculinity: karyotype-based (unisexual males), drought-induced cosexuals, or chemically induced cosexuals. Created with BioRender.com.

Bearing in mind that the cannabis industry relies heavily on the production of feminized seeds, and that male-to-male competitive dynamics may appear during the pollen grain stage of the plant life cycle, we sought to explore how characteristics associated with pollen production vary across *C. sativa*’s reproductive phenotypes (Figure 1). Using our previously validated methods for measuring pollen production [45] and pollen fitness [46], we asked: do unisexual males and cosexual plants differ in their pollen characteristics? And does the route to cosexuality, e.g., naturally occurring cosexuality in response to environmental stress, and induced cosexuality in response to chemical treatment, impact this relationship?

## 2. Results

### 2.1. Phenology

**Figure 2:**
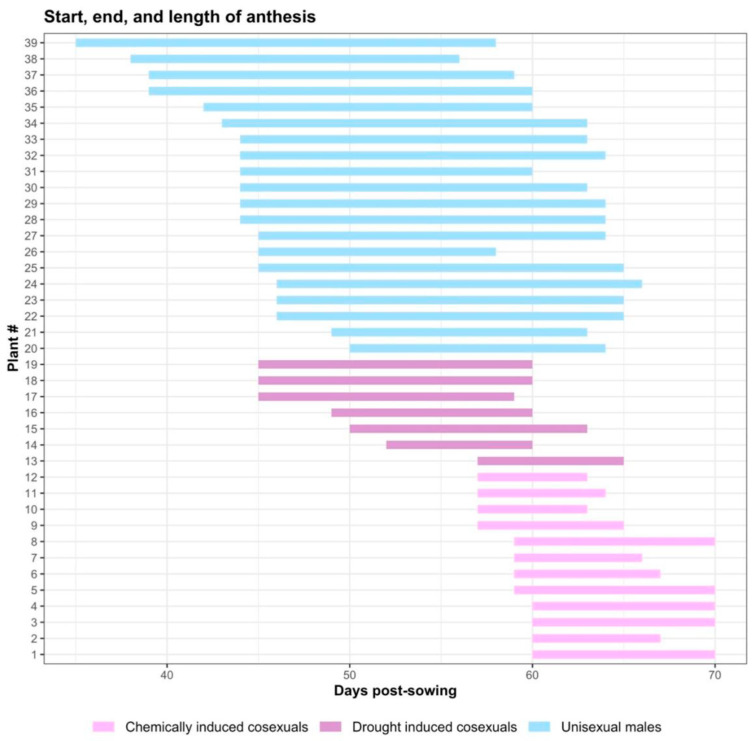
Flowering phenology of unisexual males, drought-induced cosexual plants, and chemically induced cosexual plants. Unisexual males flowered significantly earlier (F_2,36_ = 79.52, *p* < 0.001) and longer (F_2,36_ = 68.61, *p* < 0.001) than cosexual plants. The end of the flowering period differed significantly (F_2,36_ = 15.21, *p* < 0.001) between chemically induced cosexuals and both unisexual males and drought-induced cosexuals.

**Figure 3:**
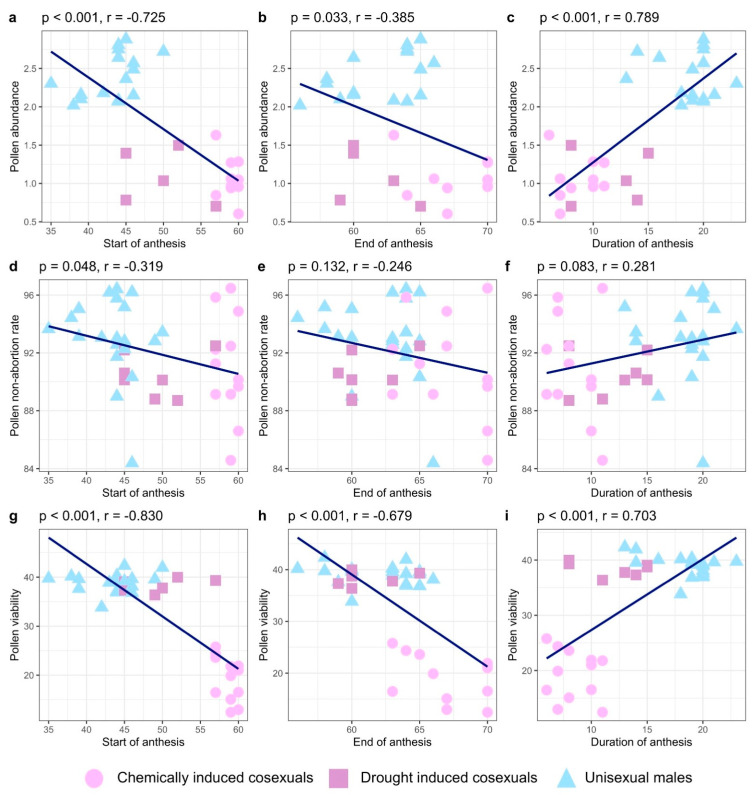
Correlations between pollen characteristics (pollen abundance, non-abortion rate, and viability) and related phenological traits (stared, duration, and end of anthesis) across three reproductive phenotypes. Blue lines indicate the direction of association.

On average, flowering occurred 5.4 days (±3.6) earlier and lasted an extra 6.6 days (±2.9) in unisexual males relative to drought-induced cosexual plants (Figure 2). Similarly, chemically induced cosexual plants flowered later than both unisexual males and drought-induced cosexual plants by 5.5 days (±2.9); though the male flowering period of this group was experimentally manipulated via chemical treatment and thus does not represent true phenological behaviour. Unisexual males flowered significantly earlier (F_2,36_ = 79.52, *p* < 0.001) than both drought-induced (Tukey’s HSD, *p* = 0.001) and chemically induced (Tukey’s HSD, *p* < 0.001) cosexual plants (Figure 3). The duration of the flowering period also differed significantly (F_2,36_ = 68.61, *p* < 0.001) between unisexual males and both drought-induced (*p* < 0.001) and chemically induced cosexuals (Tukey’s HSD, *p* < 0.001); male plants flowered longer than both drought-induced and chemically induced cosexual plants. The end of the flowering period differed significantly (F_2,36_ = 15.21, *p* < 0.001) between chemically induced cosexuals and both unisexual males (Tukey’s HSD, *p* < 0.001) and drought-induced cosexuals (Tukey’s HSD, *p* < 0.001). Broadly, flowering behaviour showed some degree of correlation to pollen characteristics (Figure 3). Pollen abundance (whole plant) was significantly correlated with the start (r = −0.72, n = 31, t_29_ = −5.66, *p* < 0.001; Figure 3a), duration (r = 0.79, n = 31, t_29_ = 6.92, *p* < 0.001; Figure 3b), and end of anthesis (r = −0.38, n = 31, t_29_ = −2.24, *p* = 0.03; Figure 3c). Pollen non-abortion rates were not significantly correlated with the duration (r = 0.28, n = 39, t_37_ = 1.78, *p* = 0.08; Figure 3e) or end (r = −0.25, n = 39, t_37_ = −1.54, *p* = 0.13; Figure 3f) of anthesis, but did display a marginally significant correlation to the start of anthesis (r = −0.32, n = 39, t_37_ = −2.04, *p* = 0.05; Figure 3d). Pollen viability was significantly correlated with the start (r = −0.83, n = 39, t_37_ = −9.05, *p* < 0.001; Figure 3g), duration (r = 0.70, n = 39, t_37_ = 6.01, *p* < 0.001; Figure 3h), and end (r = −0.68, n = 39, t_37_ = −5.6, *p* < 0.001; Figure 3i) of anthesis. Similarly to pollen yield, the start and end of anthesis were negatively correlated with pollen viability, whereas the duration of anthesis was positively correlated with pollen viability (Figure 3).

### 2.2. Pollen Abundance

**Figure 4:**
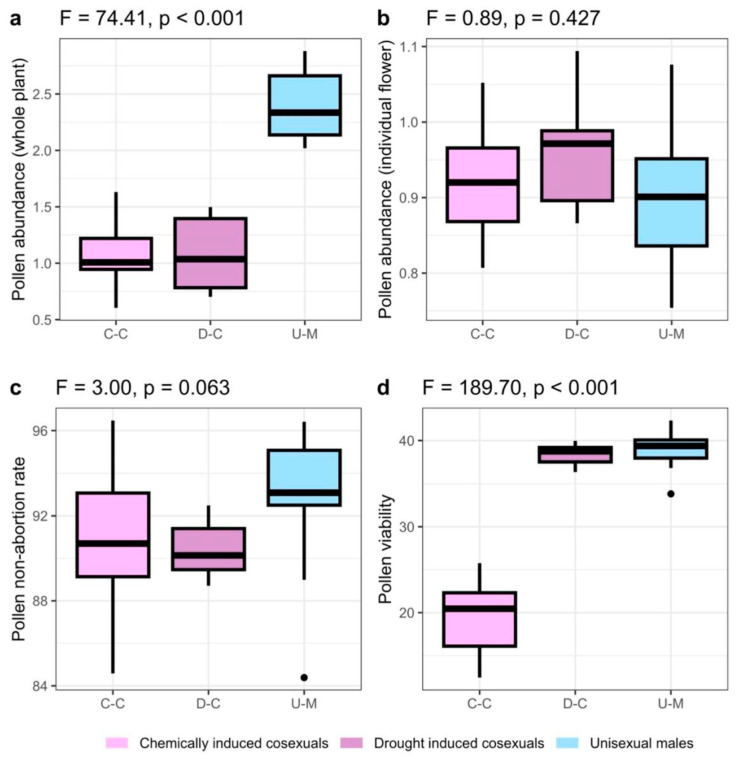
Plots of mean values (±SD) of pollen characteristics across three reproductive phenotypes. Unisexual males differed in their pollen abundance (F_2,28_ = 74.41, *p* < 0.001) from both drought-induced (Tukey’s HSD, *p* < 0.001) and chemically induced (Tukey’s HSD, *p* < 0.001) cosexual plants. Chemically induced cosexual plants differed in their pollen viability (F_2,36_ = 189.70, *p* < 0.001) from both drought-induced cosexual plants (Tukey’s HSD, *p* < 0.001) and unisexual males (Tukey’s HSD, *p* < 0.001).

Whole-plant pollen abundance differed significantly between reproductive phenotypes (F_2,28_ = 74.41, *p* < 0.001). Unisexual males differed in their pollen abundance from both drought-induced (Tukey’s HSD, *p* < 0.001) and chemically induced (Tukey’s HSD, *p* < 0.001) cosexual plants. Chemically induced and drought-induced cosexual plants did not significantly differ in their pollen abundance (Tukey’s HSD, *p* = 0.99). On average, unisexual males produced 223% more pollen than chemically induced or drought-induced cosexual plants (Figure 4a). Individual flower pollen abundance did not differ significantly between reproduction phenotypes (F_2,21_ = 0.887, *p* = 0.427; Figure 4b).

### 2.3. Pollen Non-Abortion Rates

Pollen non-abortion rates were marginally significantly different between reproductive phenotypes (F_2,36_ = 3.00, *p* = 0.06). However, there were no significant differences in pollen non-abortion rates among reproductive phenotypes in post-hoc analysis (Figure 4c). Chemically induced cosexual plants did not differ in their pollen non-abortion rates from drought-induced cosexual plants (Tukey’s HSD, *p* = 0.90), nor unisexual males (Tukey’s HSD, *p* = 0.15) and unisexual males did not differ from drought-induced cosexual plants (Tukey’s HSD, *p* = 0.11).

### 2.4. Pollen Viability

Pollen viability differed significantly between reproductive phenotypes (F_2,36_ = 189.70, *p* < 0.001). Chemically induced cosexual plants differed in their pollen viability from both drought-induced cosexual plants (Tukey’s HSD, *p* < 0.001) and unisexual males (Tukey’s HSD, *p* < 0.001). Drought-induced cosexual plants and unisexual males did not significantly differ in their pollen viability (Tukey’s HSD, *p* = 0.86). On average, unisexual males and drought-induced cosexual plants produced pollen that was 200% more viable than chemically induced cosexual plants (Figure 4d).

## 3. Discussion

Unisexual males flowered earlier and longer than both chemically induced and drought-induced cosexual plants (Figure 2). Pollen abundance and viability were strongly correlated with flowering phenology, displaying a negative relationship with the start and end of anthesis, and a positive relationship with the duration of anthesis, but the effect was driven by differences between reproductive phenotypes, rather than trade-offs between reproductive characteristics (Figure 3). Unisexual males produced significantly more pollen than cosexual plants at the whole-plant level (Figure 4a), but not the individual-flower level (Figure 4b). The protandry documented here, wherein our male plants consistently flowered earlier than our cosexual (female) plants, coincides with that seen in previous work [3,47,48]. Higher rates of pollen production in unisexual males, relative to cosexual plants, is consistent with the hypothesis that cosexual plants must allocate resources to both male and female gamete production, potentially reducing their capacity to produce either [22,49]. Noting that unisexual males produced 223% more pollen in this experiment, it is plausible that pollen production in cosexual plants may not exceed the hypothetical threshold required to disrupt an equilibrium between reproductive phenotypes in a natural population [50].

Pollen non-abortion rates did not differ significantly between unisexual males and cosexual plants (Figure 4c); however, pollen viability was substantially lower in chemically induced cosexual plants (Figure 4d). We hypothesize that this reduction in pollen germination rates could be due to the malformation of male inflorescences as a result of exposure to the colloidal silver. Colloidal silver can be used to control pathogenic fungi and bacteria in crops, but some evidence suggests that high concentrations can induce leaf necrosis [51]. Our work coincides with the literature documenting that treatment with silver has been shown to induce a cosexual phenotype in genetically female *C. sativa* plants [6,8,52,53]. Flajšman et al. [6] used colloidal silver to induce a cosexual phenotype but did not find any reduction in rates of in vitro pollen germination, potentially because they did not explicitly compare pollen performance between cosexual plants and unisexual males. Colloidal silver treatment influencing rates of pollen viability is one potential explanation that requires further investigation. Some recent work has suggested that different types of chemical treatment may induce differences in non-abortion rates [54], thus providing further justification to continue exploring these relationships.

Though these results provide interesting inferences about the dynamics of pollen production and fitness in *C. sativa*, some important limitations of the research must be considered. The use of a single cultivar and growing environment prevents us from exploring if, and how, these relationships vary across genotypic and environmental gradients. Our use of CFX-2, a photo-period sensitive variety of *C. sativa* often cultivated for its cannabidiol content, limits our ability to understand these relationships in popular commercial strains with high THC contents and dense female inflorescences [55,56]. Low sample sizes due to limitations associated with our federal research license, and specifically a low sample size of drought-induced cosexual plants, limited our statistical power. Future work should include testing multiple methods of chemically inducing a cosexual phenotype to explore if, and how, different chemical compounds impact pollen viability. Additionally, future work should include environmental manipulation, to determine if and how the external growing environment can impact measures of male reproductive traits in *C. sativa*.

## 4. Materials and Methods

### 4.1. Plant Genotype and Cultivation

**Figure 5:**
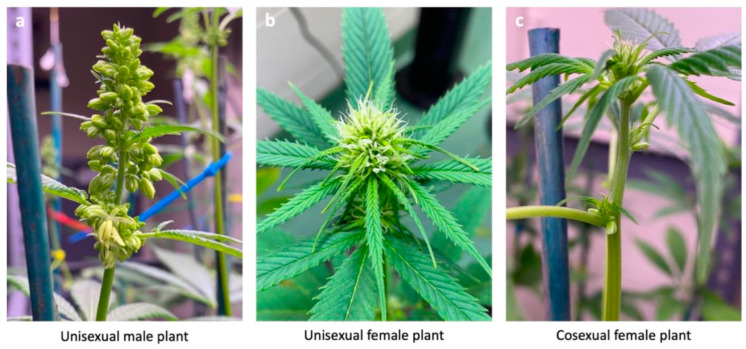
Image of plants from the experiment showing differences in floral morphology used to assign sex. Unisexual male plants (**a**) developed dense clusters of male inflorescences. Unisexual female plants (**b**) developed colas with protruding stigmas. Cosexual female plants (**c**) initially developed female flowers, and after undergoing one of two experimental treatments, later developed male inflorescences at apical branching junctions.

We used CFX-2, a dioecious variety of *C. sativa*, often cultivated for its cannabidiol content (Hemp Genetics International, Saskatoon, SK, Canada). On August 9th, 2021, we germinated 50 seeds in a two-tier terracotta germination pot (ANVÄNDBAR Sprouter; IKEA, Delft, The Netherlands), and watered them twice daily with filtered water (Milli-Q purification system #F7KA48180D, Millipore Canada Ltd., Etobicoke, ON, Canada), until they had sprouted and the radicle was >3 cm in length. We planted the germinated seedlings in circular pots (8 cm × 9.5 cm) filled with 500 mL of PRO-MIX mycorrhizae peat moss growing medium (Premier Tech, Rivière-du-Loup, QC, Canada) and placed them under fluorescent T8 bulbs (F32W, Canarm lighting and fans, Brockville, ON, Canada) for a 24-h vegetative photoperiod. After 4 weeks we shortened daylength to 12 h to induce flowering and transplanted individuals into larger circular pots (10.5 cm × 15 cm) filled with 1000 mL of PRO-MIX mycorrhizae peat moss growing medium. After we induced flowering with a shortened 12-h photoperiod, floral development occurred, and we assigned plant sex (unisexual male, unisexual female) based on development of either male and/or female inflorescences (Figure 5a,b). We moved female plants to a second growing room, isolated from the males, and staked each plant to encourage vertical growth and support delicate branches. We watered all plants thrice weekly with 250 mL of filtered water. To improve the likelihood of cosexual plants occurring in our population of female plants, we reduced watering to 150 mL (from 250 mL) thrice weekly, to simulate mild drought-like conditions. Notably, once a female plant had grown male inflorescences (Figure 5c) and was therefore tagged as a drought-induced cosexual, we returned it to a typical watering regime (250 mL, 3× per week) to minimize any risk of death. Within two weeks of imposing the shortened photoperiod and inducing mild drought-like conditions in our population of females, seven plants had developed both female and male inflorescences. To increase the sample size of cosexual plants, we randomly selected 12 remaining female plants to undergo chemical treatment [8,10] to create a second group of chemically induced cosexual phenotypes. The randomly selected 12 female plants underwent daily chemical treatment for two weeks (week 7 and 8 of the flowering growth period). Chemical treatment consisted of 15 mL of colloidal silver (Trace Minerals Research L.C., Ogden, UT, USA) diluted to 20 ppm concentration in filtered water, which we vertically sprayed onto the plants, once daily, for 14 days [6,57]. After chemical treatment, all 12 female plants developed male inflorescences and began male flowering during week 9. We note that the limited sample size and use of a single cultivar was a constraint associated with our federal research license; *C. sativa* cultivation is heavily regulated in Canada and thus impacts the scope and breadth of our research program.

### 4.2. Phenology

For each unisexual and cosexual plant included in the experiment, we noted the date of floral initiation and dehiscence. Once plants began producing immature male flowers, we monitored their development by closely examining bundles of inflorescences and looking for indicators of dehiscence, such as a visible protrusion along the lateral axis of the pollen sac followed by splitting of the sepals. We noted the commencement of male flowering as the date during which the first male inflorescence dehisced and began dispersing pollen. We identified the end of the flowering period by monitoring inflorescence development during pollen collection (described next) by noting the date on which all inflorescences had dehisced and the plant was no longer dispersing any pollen or producing new flowers. We calculated the duration of the flowering period as the number of days between the start and end of anthesis.

### 4.3. Pollen Abundance

For the duration of the flowering period, we collected pollen daily from each plant using previously validated hand-collection methods (Wizenberg et al. 2020) in 50 mL centrifuge vials. We stored pollen under room temperature conditions (22 °C ± 0.95), throughout the duration of flowering and upon conclusion of each plant’s flowering period, we suspended each pollen sample in 50 mL of distilled water and then measured the suspension’s density using visible light spectroscopy [45]. We vortexed each suspension for 30 seconds (Corning LSE vortex mixer, 230 V, product #6776; Corning Inc., Corning, NY, USA) to ensure a consistent distribution of pollen grains throughout the sample, following which we pipetted 2 mL of the suspension into a 3 mL plastic cuvette and analyzed the sample using visible light spectroscopy at a wavelength of 425 nm (Pasco wireless spectrometer and fluorometer, PS-2600; Pasco Scientific, Roseville, CA, USA).

In addition to documenting pollen production of the whole plant, we randomly selected 4 unisexual males, 2 drought-induced cosexuals, and 2 chemically induced cosexual plants to undergo individual flower analysis and thus they were excluded from whole-plant pollen production analysis. Plants selected for inclusion in individual flower analysis were closely monitored and on the day of anthesis 3 freshly dehiscent flowers were clipped and placed in 1.5 mL Eppendorf tubes. The tubes were left unsealed at room temperature for 24 h, after which all of the anthers had released their pollen. We used tweezers to carefully remove the dehiscent flowers and analyzed the resulting individual flower pollen yield using the visible light spectroscopy methods described above.

### 4.4. Pollen Non-Abortion Rates

We measured pollen non-abortion rates (the proportion of pollen grains that featured an intact generative nucleus) on the first day of anther dehiscence using a modified Alexander stain [58,59]. Before male anthesis commenced, we assembled the stain and stored it, sealed, under room temperature conditions (22 ± 0.95 °C). On the first day of pollen collection, we used a swab (Q-tips, Unilever, London, UK) to apply a small sample of pollen grains to a 75 mm × 25 mm glass slide and pipetted 20 µL of the modified stain directly onto the applied pollen sample. We then heated the slide 10 cm above a Bunsen burner for 5 s to allow the stain to set, following which we sealed the sample under a 25 mm × 25 mm glass slide cover. We left the sample to incubate at 22 °C (Fisher Scientific 6845 Isotemp Incubator 650D, Waltham, MA, USA) for 24 h, after which differential staining was visible, and we then counted the number of viable and aborted pollen grains using vertical transects at 10× magnification covering the entire length and width of the slide cover, using handheld tally counters (Uline, Pleasant Prairie, WI, USA) and a light microscope (Zeiss Primo Star Upright Light Microscope, Carl Zeiss Canada Ltd., Toronto, ON, Canada).

### 4.5. Pollen Viability

We measured pollen viability on the first day of anther dehiscence through in vitro germination, using Gaudet et al.’s liquid germination media [60], to induce rehydration and pollen tip growth in viable pollen grains. Prior to pollen anthesis, we assembled the liquid media and stored it, sealed, under room temperature conditions (22 ± 0.95 °C). On the first day of pollen collection, we used a swab (Q-tips, Unilever, London, UK) to apply a small sample of pollen to a 75 mm × 25 mm glass slide and pipetted 50 µL of the liquid media, heated to 35 °C, directly onto the applied pollen sample. We then sealed the liquid media with a 25 mm × 25 mm glass slide and incubated at 28 °C (Fisher Scientific 6845 Isotemp Incubator 650D, Waltham, MA, USA) for 24 h in a sealed Petri dish (8.5 cm × 8.5 cm × 1 cm) containing a filter paper (11 cm × 21 cm) soaked in 10 mL of water to increase the relative humidity during incubation. After incubation, germination was visible, and we then counted the number of viable pollen grains (possessing visible protrusion of a well-defined pollen tube) and the total number of pollen grains contained on the slide using vertical transects at 10× magnification covering the entire length and width of the slide cover, using handheld tally counters (Uline, Pleasant Prairie, WI, USA) and a light microscope (Zeiss Primo Star Upright Light Microscope, Carl Zeiss Canada Ltd., Toronto, ON, Canada).

### 4.6. Analysis

We conducted all analyses in R v.4.0.2. We used the *stats* package (6 April 2019, R Core Team, 2019) to complete the analysis of variance, and evaluated the parametricity of variable distributions using the residual QQ plots. We first established an experimental factor, plant sex, by assigning each plant to one of the three reproductive phenotypes based on their floral anatomy and treatment throughout the experiment (unisexual male, drought-induced cosexual plant, and chemically induced cosexual plant), and used this as a fixed effect within all models. We used the aov() function with reproductive phenotype as our experimental factor to test if the relevant response (start of anthesis, duration of anthesis, end of anthesis, pollen abundance, pollen non-abortion rate, or pollen viability) differed between subgroups, and when we saw a statistically significant effect (*p* < 0.05) we performed post-hoc analyses using Tukey’s Honest Significant Differences test, via the TukeyHSD() function, to determine how subgroups differed from one another. We used the cor.test() function included in the *stats* package to analyze product–moment correlation between pollen characteristics (pollen abundance, pollen non-abortion rate, pollen viability) and flowering phenology (start of anthesis, duration of anthesis, end of anthesis). We visualized data and relationships using the *ggplot2* package (15 June 2021, v.3.3.5), employing the base package plotting function.

## Data Availability

All data underlying the findings is available in the attached Supplementary file (S1).

## Supplementary Materials

The following supporting information can be downloaded at: https://www.mdpi.com/article/10.3390/plants12213731/s1.
